# Cranberry (*Vaccinium macrocarpon*) dietary supplementation and fecal microbiota of Wistar rats

**DOI:** 10.3934/microbiol.2021016

**Published:** 2021-06-30

**Authors:** Rayane Chettaoui, Gilles Mayot, Loris De Almeida, Patrick Di Martino

**Affiliations:** 1 Laboratoire ERRMECe, CY Cergy Paris University, 1 rue Descartes 95000 Neuville-sur-Oise, France

**Keywords:** cranberry, diet, fecal microbiota, *Escherichia coli*, *Enterococcus*, *Lactobacillus*

## Abstract

Cranberry (*Vaccinium macrocarpon*) dietary supplementation can help prevention of urinary tract infections through the supply of proanthocyanidin-type polyphenols (PAC). The main uropathogenic bacteria are members of the intestinal microbiota. A randomized cross-over experiment was done to investigate whether cranberry dietary supplementation affects concentrations of thermotolerant coliforms, *Enterococcus* spp. and *Lactobacillus* spp. in rat faeces. Thirteen rats, housed in individual cages, received successively two diets as pellets during 7 days each: a standard diet without polyphenols and the standard diet supplemented with cranberry powder containing 10.9 mg/100 g of PAC. There was a 7 days wash-out period in between with standard diet without polyphenols. Body weight and feed intake were recorded. Faeces were collected on the last day of treatment, and crushed to count the different bacterial populations using the most probable number method. Thermotolerant coliforms were grown in BGBLB tubes and on MacConkey agar. *Enterococcus* spp. were grown in Rothe and Litsky broths and on KF *Streptococcus* agar. *Lactobacillus* spp. were grown in Man Rogosa Sharpe broth. Body mass gains were not affected by cranberry supplementation. This is consistent with equal food intake, cranberry powder not providing significant energy supplement. Cranberry dietary supplementation was associated with changes in fecal concentrations of thermotolerant coliforms, and *Enterococcus* spp. in some rats, but did not induce significant changes in bacterial fecal concentrations in a global population of 13 rats. In conclusion, we did not observe any significant effect of dietary cranberry supplementation on the fecal microbiota of Wistars rats for a 7-day diet.

## 1. Introduction

The Cranberry (*Vaccinium macrocarpon*) is a fruit of the Ericaceae family. It is a round red berry native to North America, about 1 cm in diameter with high acidity and high content of tannins (polyphenols). Numerous scientific studies realised *in vivo*, *ex vivo* and *in vitro* have shown that food consumption of cranberry can be associated with a decrease in the incidence of urinary tract infections (UTI) in women, can reduce the concentration of bacteria in the urine of women, and that the presence of cranberry or some cranberry components decrease the adhesion of uropathogenic strains of *Escherichia coli* to urinary epithelial cells [1]–[14]. Some of the mechanisms by which cranberry protects the urinary tract from bacterial infection have been elucidated, but others remain to be demonstrated [15]. A specific group of cranberry components corresponding to a particular type of tannin called proanthocyanidin-type polyphenols (PAC) type A are involved in the bacterial adherence inhibition activity [16]. Type A PAC act on type P pili of uropathogenic *E. coli* (UPEC) strains by significantly reducing their ability to attach to the surface of cells lining the urinary system [16]. Soluble PACs are needed to obtain an anti-adherence effect against UPECs *in vitro* and *ex vivo* [17]. B-ring substituted flavones and flavonols act on UPEC type 1 pili by reducing their attachment to bladder epithelial cells [13]. Metabolites of PACs and flavan-3-ols produced *in vivo* after dietary intake of cranberry or other food (tea, chocolate, etc.) inhibit *in vitro* UPEC adherence to bladder epithelial cells [18],[19]. In addition to *E. coli*, other intestinal microbiota species such as *Enterococcus faecalis* are also responsible for UTI [20]. Cranberry extracts have been shown to inhibit both planktonic and biofilm growth of *E. coli* and *E. faecalis in vitro* [21],[22]. The intestinal tract is a reservoir for uropathogenic bacteria towards an ascending pathway of fecal-perineal-urethral infection [23]. Interactions between components and/or metabolites of the cranberry and the intestinal microbiota could be involved in the prevention of urinary tract infections [24],[25]. While numerous studies on the effects of cranberry consumption on the incidence of UTI are available, there is little data in the literature on the impact of this dietary consumption on the intestinal microbiota or fecal flora [26]–[28]. Moreover, PAC can act on the interactions between pathogenic *E. coli* and the intestinal epithelium [29].

The objective of the study was to investigate whether intake of cranberry powder affects the fecal flora in Wistar rat. We analysed three types of flora, thermotolerant coliforms and *Enterococcus* spp., both involved in UTI, and *Lactobacillus* spp., non-pathogenic bacteria capable of migrating in the genital tract and forming the vaginal flora, some strains harbouring probiotic activity in the intestinal tract.

## 2. Materials and method

### 2.1. Animals and diets

Thirteen male *Rattus norvegicus* Wistar rats (Centre d'élevage d'Ardenay, Bray-Lu, France) weighting approximately 228 g were housed in the conventional animal facility of the ‘département génie biologique’ of the ‘Institut Universitaire de Technologie’ of Cergy-Pontoise. All animal care and experimental procedures complied with the European Union legislation on the protection of animals used for scientific purposes (2010/63/EU). Animal facility are approved by the French veterinary department (A95001). Rats were housed in individual cages, subjected to a 12 h light/dark cycle at a temperature of 20 ± 2 °C. All animals had *ad libitum* access to food and water. For 7 days, the rats were acclimatized in conventional cages and fed with the standard diet (diet C) consisting of pellets. The diet C contained corn starch (39.75%), casein (18%), maltodextrine (14.2%), sucrose (11%), soya oil (7%), cellulose (5%), mineral AIN 93 (3.5%), vitamin AIN93 (1%), cystine (0.3%), bitartrate choline (0.25%) (SAFE, Augy, France). The diet used was a purified AIN-93-based diet, which contained very limited amounts of any polyphenolic compounds.

The study was a randomized cross-over study consisting of a succession of two diets of 7 days each with collection of faeces. A treatment duration of 5 days has already been used successfully in a previously published similar study [25]. The rats were individually placed in a metabolism cage for the last 4 days of each diet (Tecniplast, Decines-Charpieu, FRANCE). The metabolism cages make it possible to collect urine and faeces independently without any risk of alteration or cross-contamination of urine and faeces during the process. Each rat took the two regimens successively in a randomly order, with a washout period of at least 7 days between each regimen change as previously described [6],[30]. Rats ate diet C during the wash-out period. The diet supplemented with cranberry (diet A) corresponded to the diet C to which Urell^©^ food supplement powder (Pharmatoka, Rueil-Malmaison, France) has been added so as to obtain a final concentration of 10.9 mg per 100 g of PAC. The Urell^©^ food supplement powder has been incorporated into the food by the pellet manufacturer during the production process (SAFE, Augy, France). Food intake (taking waste into account) has been averaged over the last 3 days in the metabolism cage for each rat. The body mass gain over the 7 days of treatment was determined by weighing each rat on the first day and last day of each diet. Faeces were collected on the last day of treatment for each diet.

### 2.2. Faeces analysis

About 2 g of fresh faeces were transferred to a 50 mL centrifuge tube and diluted with 10 mL Phosphate Buffered Saline containing 20% glycerol. The samples were homogenized by adding 10 glass beads and vortexing the whole for 3 minutes. The samples were then centrifuged for 5 min at 300 g in a free rotor centrifuge in order to pellet beads and large particles present in the faeces. The supernatant was collected and frozen at −20 °C until used. The supernatants obtained after treatment were analysed for their bacterial content according to the method known as the most probable number (MPN) using the culture media described below. Three populations were targeted: thermotolerant coliforms (presumed *E. coli*), *Enterococcus* spp. and *Lactobacillus* spp. This MPN method was derived from the ISO 7218:2007 standard.

Before dilution, the samples were thawed for 2 hours at + 4 °C. The serial dilutions were carried out in sterile physiological serum. Samples from the same rat (diet C and diet A) were analysed on the same day. We ensured that freezing had no impact on the bacterial count by comparing the results of the count before and after freezing (data not shown).

The enumeration of thermotolerant coliforms was derived from the ISO 7251:2005 standard. The enumeration was carried out with the Brilliant Green Bile Lactose Broth (BGBLB) medium (Fisherbrand, Illkirch, France) incubated at 44 °C for 24 h. Confirmation of the presence of *E. coli* in BGBLB positive tubes was performed by inoculating Mac Conkey agar plates (Merck, Fontenay-sous-Bois, France). After incubation for 24 h at 44 °C, six lactose degrading colonies were identified using Api 20E biochemical identification tests (BioMérieux, Lyon, France). The enumeration of *Enterococcus* was carried out by successive seeding of Rothe broth then of Litsky broth (Biokar, Beauvais, France) incubated 48 h at 37 °C. Confirmation of the presence of *Enterococcus* spp. in Litsky broth was obtained by isolation on Streptococcal Kenner fecal agar plates supplemented with 1% TTC (VWR Chemicals, Fontenay-sous-Bois, France). Four bacterial isolates obtained were identified using Api STREP biochemical identification tests (BioMérieux, Lyon, France). The enumeration of *Lactobacillus* spp. was done with the Man Rogosa Sharpe broth (VWR Chemicals, Fontenay-sous-Bois, France) incubated until 3 days at 37 °C.

### 2.3. Statistical analysis

All values are given as means ± SE. Statistical analyses were performed using XLSTAT software, version 2013.5.09 (Addinsoft, Paris, France). Values of *P* < 0.05 were considered signiﬁcant for all analyses. The signiﬁcance of the diet effect was analysed with a signed Wilcoxon test for paired data.

## 3. Results and discussion

The composition and metabolism of the gut microbiome are directly affected by dietary intake [31]. By controlling diet, it is possible to modify this microbiome by promoting the growth of certain bacteria that have beneficial effects on health, and by decreasing the concentration of bacteria with harmful effects [32]–[34]. In this study, we determined whether dietary cranberry supplementation altered fecal concentrations of two types of flora involved in urinary tract infections, thermotolerant coliforms and *Enterococcus* spp., and a type of flora beneficial to health, *Lactobacillus* spp.

### 3.1. Food consumption and evaluation of body mass

In a first step, the impact of dietary cranberry supplementation on the growth of rats was determined. The body mass gain during the 7 days of treatment was not affected by PAC supplementation (Table 1). Indeed, rats supplemented with cranberry (diet A) had a growth of 46.8 ± 4.2 g in 7 days and rats not supplemented (diet C) of 49.4 ± 3.0 g over the same period. This equivalent growth is explained by an almost identical food intake between the two groups and by the fact that PAC do not provide a significant energy supplement. Thus, the rats consumed 26.9 ± 1.2 g of pellets/day and 26.1 ± 0.9 g of pellets/day in the non-supplemented rats (diet C) and in the supplemented rats (diet A), respectively. This corresponded to an average intake of 2.85 ± 0.09 mg of PAC/day for rats supplemented with cranberry (diet A). The amount of PAC ingested by each rat was equivalent to the amount recommended in humans. Indeed, a factor of 20 is generally applied between humans and rats for nutritional intake, i.e., 57 ± 1.8 mg of PAC/day after calculation [35]. This calculated value was close to 36 mg of PAC/day, for the consumption of cranberry-based products, recommended in humans in France to ‘help reduce the fixation of certain *E. coli* bacteria on the walls of the urinary tract’ [36].

**Table 1.** Food consumption and evaluation of body mass of the rats during the experimental period (n = 13).

	Diet C	Diet A
Proanthocyanidin-type polyphenols (mg/day)	0	2.85 ± 0.09
Body mass gain (g/7 days)	49.4 ± 3.0	46.8 ± 4.2
Food intake (g/day)	26.9 ± 1.2	26.1 ± 0.9

Values are means ± SE. Diet C was a standard diet without polyphenols. Diet A was a standard diet supplemented with cranberry powder containing 10.9 mg/100 g of proanthocyanidin-type polyphenols. The body mass gain over the 7 days of treatment was determined by weighing each rat on the first day and last day of each diet. Food intake (taking waste into account) has been averaged over the last 3 days in the metabolism cage for each rat.

### 3.2. Microbiological analyses of rat faeces

**Figure 1.**
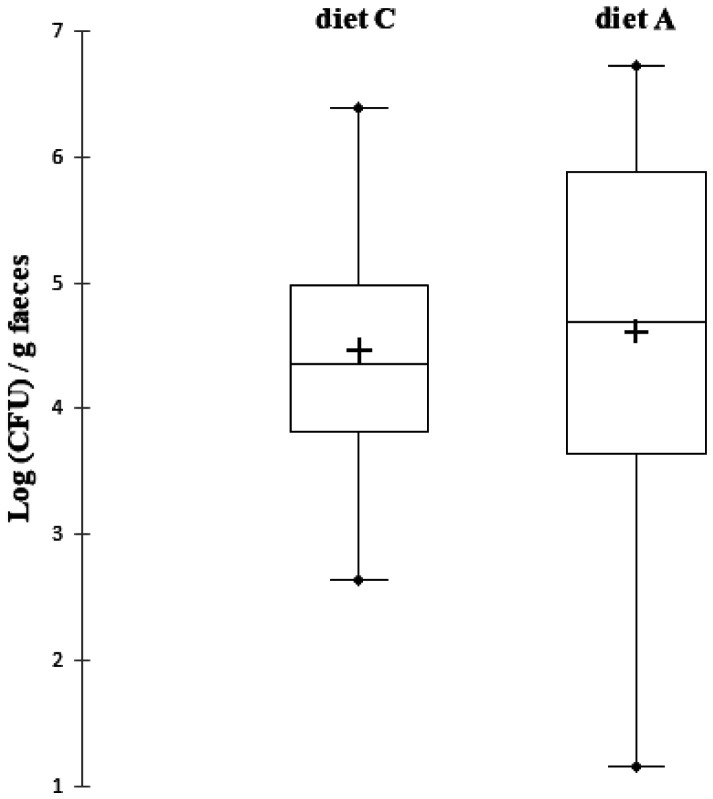
Box-plot representation of thermotolerant coliforms counts after the 7 days experimental period with two different diets in Wistar rats (n = 12). Diet C was a standard diet without polyphenols. Diet A was a standard diet supplemented with cranberry powder containing 10.9 mg/100 g of proanthocyanidin-type polyphenols. The middle region of each box plot (box body) covers 50% of the animals. The median values are presented in black lines inside the box bodies. The upper black dots present the maximum and the lower black dots present the minimum. The mean values are presented in crosses inside the box bodies. The signiﬁcance of the diet effect was analysed with a signed Wilcoxon test for paired data.

The count of thermotolerant coliforms showed a very high variability in bacterial concentration between rats (Figure 1, Table 2). One rat was excluded from the analysis of thermotolerant fecal coliform counts because its bacterial concentration was almost zero. A difference of at least 1 Log CFU/g was considered to be a quantitative variation of the corresponding bacterial population [37]. For rats not supplemented with cranberry (diet C), the concentration of thermotolerant coliforms varied from 2.64 Log (CFU)/g of faeces to 6.39 Log (CFU)/g of faeces with a median value of 4.36 Log (CFU)/g of faeces. The variability in bacterial concentration between rats was even greater for supplemented rats (diet A). Indeed, the bacterial concentration varied from 1.16 Log (CFU)/g of faeces to 5.99 Log (CFU)/g of faeces with a median value of 4.69 Log (CFU)/g of faeces. After analysis of the rat-by-rat results, three populations of rats were observed for their sensitivity to the consumption of cranberry. For four out of 12 rats (rats B, E, H, J), the bacterial concentration of thermotolerant fecal coliforms increased after consumption of cranberry. For two rats (I, L), the average concentration of thermotolerant fecal coliforms decreased after consumption of cranberry. For the other six rats, the concentration was not changed by diet. Overall, on all rats, there was no significant difference between the two groups with different diets and therefore cranberry supplementation did not affect the fecal bacterial concentration in thermotolerant coliforms. Confirmation of the presence of *E. coli* in BGBLB positive tubes was performed by inoculating Mac Conkey agar plates, and analysing lactose degrading colonies using Api 20E biochemical identification tests. The six bacterial isolates had the same biotype corresponding to the API code 5 144 572, which made it possible to identify them as *E. coli* with a confidence percentage of 99.5%.

**Table 2.** Thermotolerant coliforms counts after the 7 days experimental period with two different diets in Wistar rats (n = 12).

Rat	Bacterial concentration Log (CFU)/g faeces	
	Diet C	Diet A
A	4.98	5.84
B	2.64	3.64
C	4.06	3.30
D	4.87	4.30
E	4.67	5.99
F	3.65	3.65
G	4.99	4.85
H	3.86	6.73
I	3.68	1.16
J	4.05	5.39
K	6.39	5.99
L	5.73	4.53
Mean	4.46	4.61

Bacterial counts were determined with the most probable number method (ISO 7251:2005 standard). Diet C was a standard diet without polyphenols. Diet A was a standard diet supplemented with cranberry powder containing 10.9 mg/100 g of proanthocyanidin-type polyphenols.

**Figure 2.**
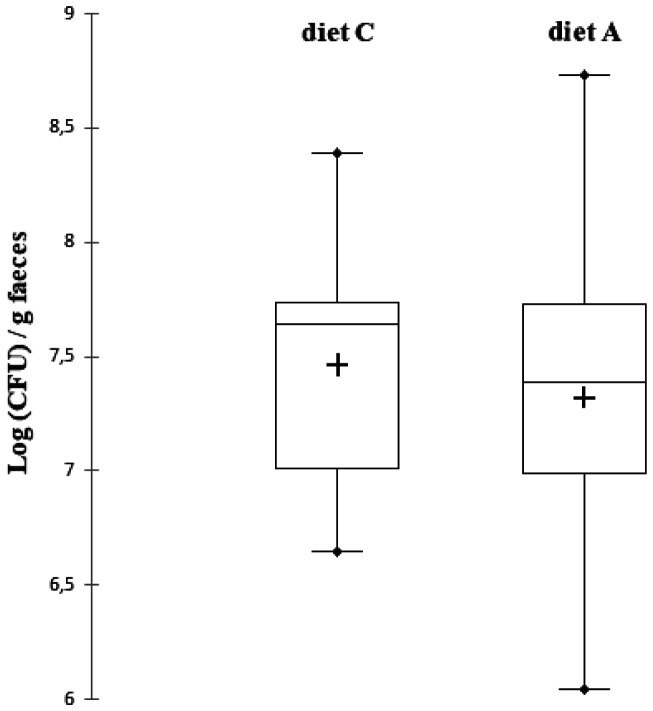
Box-plot representation of *Enterococcus* spp. counts after the 7 days experimental period with two different diets in Wistar rats (n = 13). Diet C was a standard diet without polyphenols. Diet A was a standard diet supplemented with cranberry powder containing 10.9 mg/100 g of proanthocyanidin-type polyphenols. The middle region of each box plot (box body) covers 50% of the animals. The median values are presented in black lines inside the box bodies. The upper black dots present the maximum and the lower black dots present the minimum. The mean values are presented in crosses inside the box bodies. The signiﬁcance of the diet effect was analysed with a signed Wilcoxon test for paired data.

The enumeration of fecal *Enterococcus* spp. also showed certain variability in bacterial concentration between rats (Figure 2, Table 3). Although *Enterococcus* spp. generally represent less than 1% of the intestinal flora [38], *Enterococcus* spp. and in particular *E. faecalis* and *E. faecium* are found in high concentrations in human faeces, generally between 10^4^ and 10^6^ bacteria per gram [39]. In the present study, high fecal enterococcal concentrations were measured in rats, in the order of 10^8^ CFU per gram. For rats not supplemented with cranberry (diet C), the concentration of fecal *Enterococcus* spp. varied from 6.64 Log (CFU)/g of faeces to 8.39 Log (CFU)/g of faeces with a median value of 7.74 Log (CFU)/g. The variability in bacterial concentration between rats was slightly increased for supplemented rats (diet A). Indeed, the bacterial concentration varied from 6.04 Log (CFU)/g of faeces to 8.73 Log (CFU)/g of faeces with a median value of 7.39 Log (CFU)/g for this diet A. By analysing the results rat by rat, cranberry supplementation had almost no effect on the bacterial concentration of fecal *Enterococcus* spp. An increase in the fecal enterococcal concentration of at least 1 Log (CFU)/g was observed only for rats E and J and a decrease in the same proportions only for rat L. For all the other rats, the fecal concentration of *Enterococcus* spp. remained unchanged regardless of diet. Thus, as with the bacterial concentration of thermotolerant coliforms, when we analysed the whole population of rats, there was no significant difference in the concentration of fecal *Enterococcus* spp. between the two groups with different diets. Confirmation of the presence of *Enterococcus* spp. in Litsky broth was obtained by inoculating Streptococcal Kenner fecal agar plates supplemented with 1% TTC, and analysing isolated colonies using Api 20 Strep biochemical identification tests. Among the four bacterial isolates tested, three biotypes corresponding to an identification as *Enterococcus durans* were obtained. Two isolates had the same biotype corresponding to the API code 7 253 410 (*E. durans*, identification confidence percentage of 81.8%), one isolate had the biotype corresponding to the API code 7 363 410 (*E. durans*, identification confidence percentage of 96.5%), and one isolate had the biotype corresponding to the API code 7 113 410 (*E. durans*, identification confidence percentage of 96.1%). This showed that, despite belonging to the same species, there was some diversity among *Enterococcus* isolates from rat faeces. In veterinary medicine, it is common to identify the presence of *Enterococcus* spp. in urine cultures of dogs and cats [40].

**Table 3.** *Enterococcus* spp. counts after the 7 days experimental period with two different diets in Wistar rats (n = 13).

	Bacterial concentration Log (CFU)/g faeces	
Rat	Diet C	Diet A
A	7.72	7.10
B	7.64	6.99
C	7.40	6.65
D	7.74	7.39
E	7.01	8.73
F	6.65	6.99
G	6.99	6.98
H	7.38	7.39
I	7.77	7.73
J	6.64	7.73
K	7.73	7.73
L	7.99	6.04
M	8.39	7.74
Mean	7.46	7.32

Bacterial counts were determined with the most probable number method (ISO 7218:2007 standard). Diet C was a standard diet without polyphenols. Diet A was a standard diet supplemented with cranberry powder containing 10.9 mg/100 g of proanthocyanidin-type polyphenols.

**Figure 3.**
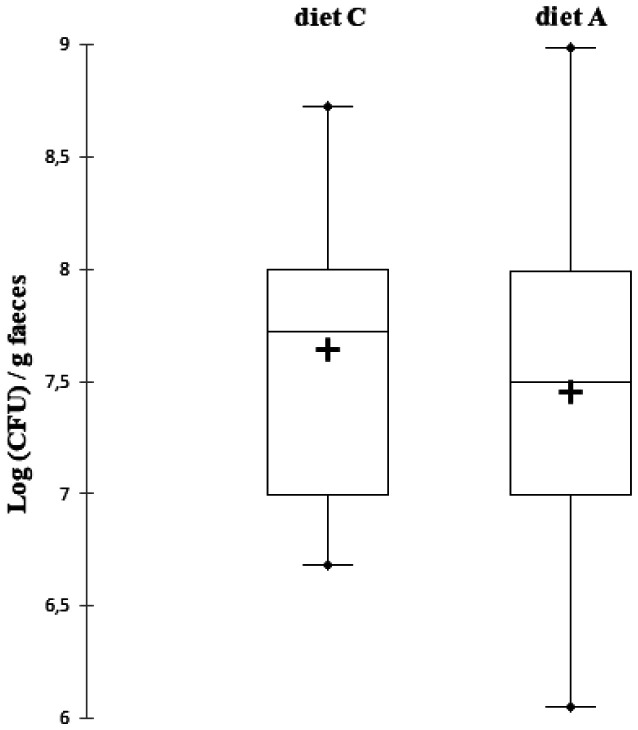
Box-plot representation of *Lactobacillus* spp. counts after the 7 days experimental period with two different diets in Wistar rats (n = 13). Diet C was a standard diet without polyphenols. Diet A was a standard diet supplemented with cranberry powder containing 10.9 mg/100 g of proanthocyanidin-type polyphenols. The middle region of each box plot (box body) covers 50% of the animals. The median values are presented in black lines inside the box bodies. The upper black dots present the maximum and the lower black dots present the minimum. The mean values are presented in crosses inside the box bodies. The signiﬁcance of the diet effect was analysed with a signed Wilcoxon test for paired data.

The enumeration of fecal *Lactobacillus* spp. showed little variability in bacterial concentration between rats (Figure 3, Table 4). For diet C, the maximum was 8.73 Log (CFU)/g of faeces and the minimum of 6.68 Log (CFU)/g with a median value of 7.73 Log (CFU)/g of faeces. For diet A, the median value was 7.57 Log (CFU)/g of faeces, the maximum obtained was 8.98 Log (CFU)/g and the minimum of 6.05 Log (CFU)/g. Looking at the results of the rat-by-rat *Lactobacillus* count, there was no change in the fecal concentration of *Lactobacillus* spp., whatever the diet. There was no significant difference of the fecal *Lactobacillus* spp. concentrations between the two groups with different diets when considering all rats. *Lactobacillus* spp. have been previously counted in the faeces of humans, hens, and pigs, at concentrations of 5.5 × 10^10^ (CFU)/g of faeces, 4.7 × 10^8^ (CFU)/g of faeces, and 9.7 × 10^8^ (CFU)/g faeces, respectively [41]. In rats, we systematically observed the presence of *Lactobacillus* spp. in the faeces and measured fecal concentrations in the order of 10^8^ (CFU)/g of faeces. In humans, only 75% of individuals have *Lactobacillus* spp. in the faeces [42]. *Lactobacillus* spp. are never pathogenic but they are members of the intestinal microbiota. The enumeration of *Lactobacillus* spp. in the presence of PAC allowed us to observe the absence of impact of PAC on the fecal concentration of non-pathogenic bacteria capable also of migrating in the urogenital tract and of forming the vaginal flora. In addition, certain strains of *Lactobacillus* spp. are known to have probiotic activity [43] and there are commercially available food supplements containing *Lactobacillus* spp. probiotics alone, or, conjugated to cranberry. Some of these food supplements have shown effectiveness in preventing recurrent urinary tract infections in women [43],[44]. Certain probiotic strains of *Lactobacillus* spp. inhibit the adhesion of uropathogenic strains of *E. coli* and *E. faecalis* to epithelial cells of the bladder *in vitro* [45].

**Table 4.** *Lactobacillus* spp. count after the 7 days experimental period with two different diets in Wistar rats (n = 13)

	Bacterial concentration Log (CFU)/g faeces	
Rat	Diet C	Diet A
A	8.38	7.50
B	6.99	6.29
C	7.00	7.00
D	8.00	7.65
E	7.75	8.38
F	6.99	6.05
G	7.38	7.98
H	7.72	7.39
I	6.68	6.29
J	7.99	7.99
K	7.39	7.39
L	8.73	8.98
M	8.39	8.00
Mean	7.64	7.45

Bacterial counts were determined with the most probable number method (ISO 7218:2007 standard). Diet C was a standard diet without polyphenols. Diet A was a standard diet supplemented with cranberry powder containing 10.9 mg/100 g of proanthocyanidin-type polyphenols.

Analysis of faeces with enumeration of fecal coliforms and *Enterococcus* spp. showed a variability in bacterial concentration between rats for each of these 2 florae. In the case of *Lactobacillus* spp., the variability between rats was weaker. Regardless of the population measured, the variability tended to increase following cranberry supplementation, the minimum and maximum values being decreased and increased, respectively. The median value of fecal bacterial concentration increased slightly for thermotolerant coliforms while it decreased slightly for *Enterococcus* spp. and *Lactobacillus* spp. This illustrates that the effects of cranberry supplementation are not the same on the different fecal flora. Thus, some bacterial species may be sensitive to changes in the diet and others may not. In humans, there are differences between the fecal flora of different individuals and, for the same individual, a change in diet induces varied quantitative fluctuations depending on the bacterial species analysed [46]. Differences in the composition of the gut microbiome may account for inter-individual differences in the efficacy of dietary cranberry supplementation in the prevention of UTIs. For example, in a model of obese mice stuffed with a diet rich in sugars and fats, food supplementation with cranberry extract induces an increase in the proportion of bacteria of the genus *Akkermansia* capable of degrading mucins [26]. The addition of cranberry to an animal-based diet has been shown to partially restore alterations in the composition and function of the gut microbiota associated with this regimen [25]. Thus, cranberry is thought to prevent dysbiosis of the intestinal microbiota.

## 4. Conclusions

In this randomized cross-over study, we investigated whether cranberry dietary supplementation can affect fecal concentrations of thermotolerant coliforms, *Enterococci* spp. and *Lactobacilli* spp. in Wistar rats. Despite the observation of variations in fecal bacterial concentrations for certain rats, we did not observe any significant modification of the fecal concentrations of the different flora studied, in connection with the food consumption of cranberry. This could be linked to the food matrix used to deliver the cranberry components [47]. A similar new study should be conducted with a larger number of rats and a different food matrix to confirm this result.

## Acknowledgments (All sources of funding of the study must be disclosed)

This study has been supported in part by a grant from the ‘Fondation Université de Cergy-Pontoise’, and by CNRS GDR 2088 ‘BIOMIM’. We thank the ‘département génie biologique’ of the ‘Institut Universitaire de Technologie’ of Cergy-Pontoise for the access to its conventional animal facility.
